# 

**Published:** 1886-05

**Authors:** 


### EDITORIAL.

### To State and Local Dental Societies.

Remember that every local society which has adopted substantially the code of ethics of the American Dental Association is entitled to one delegate for every five members. Appoint your delegates soon and let all unite in making this the largest and most profitable meeting of the Association every yet held.

J. N. CROUSE, Chairman Executive Committee.

Chicago, April 23, 1886.

### Meeting.

The annual meeting of the Mad River Valley Dental Society will be held in the parlor of the Phillip's House, in Dayton, on Tuesday, the 18th of May, beginning at 10 o'clock A. M., and continue through the day.

The following list of subjects will be presented for discussion:

1. The Advantages of making Rubber Plates before attaching the Teeth.

2. Every Day Practice.

3. The Treatment of Pulpless and Gaseous Teeth.

4. The Advantage of Amalgam and Oxyphosphate Combined as a filling material.

There will be papers read on these and other subjects. This will doubtless be an interesting meeting, as the time will be almost wholly devoted to the consideration of the professional topics brought forward. This society has always done just what all merely local societies should do, viz.: ignore professional politics, and attend to strictly scientific and practical matters.

It is hoped that there will be a full attendance, and that every one will come prepared to add something to the interest of the occasion.

### American Medical Association.

This body holds its annual meeting in St. Louis, beginning Tuesday, May 4th, and continuing four days. This is a national association, and brings together the leading men of the medical profession of the United States. It exercises a large influence upon the medical profession in all its departments, even those practicing specialities, are, or should be deeply interested in its doings.

The present meeting will be one of the most important ever held; a wider interest is now entertained in regard to it than ever before; and there are some matters to be adjusted and decided of very great importance.

A section on *Oral and Dental Surgery* was organized by this body a few years ago, and was put upon the same footing as the other sections. The dentists have not made this opportunity as fruitful as they might, and as they ought; properly utilized, it will lead to more intimate relationship between physicians and dentists. This would undoubtedly be mutually helpful, and especially so to the latter. It is very desirable that this section in the future be more fully attended, and better effort made for its success.

### The American Dental Association.

The next annual meeting of this body will be held at Niagara Falls. It has been with considerable difficulty that this matter has been settled. Chicago and San Francisco had strong advocates, but neither enough to decide the question. So Niagara was accepted as a compromise, and a very proper one it is too.

When the selection of the place of the next meeting was left in the hands of the Executive Committee, it was supposed they would readily decide upon a place. But the question was too much for them, and they found it necessary to call for a good deal of outside help, and after long and laborious effort, a decision was reached, a place chosen, and it is to be hoped that all are happy, and that there will be a large attendance of the members and the profession, and may it be one of the most successful meetings ever held.

### THE DENTAL REGISTER,

A MONTHLY MAGAZINE DEVOTED TO THE INTERESTS OF THE

**DENTAL PROFESSION.**

*Terms Reduced to $2.00 Per Year. Single Copies, 25 Cents.*

**EDITED BY PROF. J. TAFT, D.D.S.**

—AND—

**Published by SAM'L A. CROCKER & CO., Ohio Dental and Surgical Depot.**

The volume commences in January and closes in December.

The REGISTER being issued monthly is always fresh, therefore Indispensable to the practitioner who desires to keep posted up with the new inventions and improvements which are daily developed. Neither expense nor effort will be spared to give value and variety to its contents. Articles will be illustrated with well executed engravings whenever they will add to the interest or assist to explanation.

Its extensive circulation and frequency of issue make it one of the BEST DENTAL ADVERTISING MEDIUMS in the United States.

All subscriptions must begin with January or July.

Local or special notices, five lines or less, will be inserted for $3 each insertion; over five lines, 50 cts. per line.

All advertising bills payable in advance, or at furthest, after the issue of the first number containing the advertisement. All transient advertisements to be paid for in advance.

☞CONTRIBUTIONS SOLICITED.

Exclusively Editorial Communications should be addressed to the Editor.

The latest number of the REGISTER may be ordered with or without other goods at any time, and all letters should be addressed to

**SAM'L A. CROCKER & CO., Ohio Dental and Surgical Depot, Cincinnati, O.**

